# Gut microbiota-dependent adaptor molecule recruits DNA methyltransferase to the TLR4 gene in colonic epithelial cells to suppress inflammatory reactions

**DOI:** 10.3389/fmolb.2022.1005136

**Published:** 2022-10-21

**Authors:** Hikari Narabayashi, Chiharu Koma, Kazuaki Nakata, Mion Ikegami, Yusuke Nakanishi, Jun Ogihara, Masato Tsuda, Akira Hosono, Shigemasa Hanazawa, Kyoko Takahashi

**Affiliations:** ^1^ Department of Applied Biological Science, College of Bioresource Sciences, Nihon University, Fujisawa, Japan; ^2^ Department of Chemistry and Life Science, College of Bioresource Sciences, Nihon University, Fujisawa, Japan; ^3^ Department of Food Bioscience and Biotechnology, College of Bioresource Sciences, Nihon University, Fujisawa, Japan

**Keywords:** gut microbiota, intestinal epithelial cell, TLR4, DNA methyltransferase, RBM14

## Abstract

The intestine is inhabited by a large number of commensal bacteria that are immunologically non-self, potentially causing inflammation. However, in a healthy intestine, inflammation is strictly controlled at low levels to maintain homeostasis. We previously reported that the gut microbiota induce DNA methylation of the gene encoding Toll-like receptor (TLR) 4, a pattern recognition receptor that recognizes lipopolysaccharides of gram-negative bacteria, in colonic epithelial cells, suggesting its role in controlling intestinal inflammation. However, there remains a question of how gut microbiota cause methylation of only specific genes including TLR4, despite the fact that DNA methyltransferase (DNMT) is common to all genes targeted for methylation. Here, we identified RBM14 as an adaptor molecule that recruits DNMT to the TLR4 gene. RBM14 was shown to bind DNMT3 and be expressed at significantly higher levels in an intestinal epithelial cell (IEC) line with hypermethylated TLR4 gene than in an IEC line with hypomethylated TLR4 gene. In addition, RBM14 interacted with DNA regions of the TLR4 gene, and knockdown of RBM14 suppressed DNA methylation of the TLR4 gene in IECs. Furthermore, RBM14 expression was higher in colonic epithelial cells of conventional mice than in those of germ-free mice. Collectively, these results indicate that the gut microbiota induce methylation of the TLR4 gene in colonic epithelial cells by upregulating RBM14, which can recruit DNMT3 to the gene. The regulation of adaptor molecules such as RBM14, which bind to specific target genes and recruit DNMT, can explain, at least in part, how gut microbiota contribute to the maintenance of intestinal homeostasis through epigenetic control of specific gene expression in IECs.

## 1 Introduction

The intestinal tract is inhabited by as many as 40 trillion bacteria, which is greater than the 30 trillion cells consisting human body (Sender et al., 2016). In recent years, the physiological effects of these commensal bacteria on the host have attracted considerable attention. Numerous reports have shown a correlation between the composition of the gut microbiota and the incidence of various diseases (Miyauchi et al., 2022; Needham et al., 2022; Park et al., 2022; Rahman et al., 2022; Sadrekarimi et al., 2022; Tan et al., 2022), allowing the essential role of the gut microbiota in the maintenance of human health to now be widely understood. In addition, the cellular and molecular mechanisms by which the gut microbiota regulate host immune, nervous, and endocrine systems are being rapidly elucidated (Nemet et al., 2020; Schluter et al., 2020; Stefan et al., 2020; Wei et al., 2021; Bostick et al., 2022; Brodin, 2022; Cook and Mansuy-Aubert, 2022; Erttmann et al., 2022; Paik et al., 2022).

Despite the presence of the largest immune system in the body, the intestine allows a substantial number of commensal bacteria to exist without exclusion. If the intestinal immune system recognizes and reacts to large numbers of commensal bacteria in the same way as invading pathogenic bacteria, it triggers severe inflammatory reactions. However, although commensal bacteria are immunologically non-self, such intense inflammation does not occur in healthy intestines, indicating the presence of specific mechanisms to prevent it and establish a symbiotic relationship between commensal bacteria and the host immune system. Dysbiosis due to disturbances in the composition of gut microbiota results in disorders of these mechanisms, which increases the risk of developing or exacerbating inflammatory conditions, such as inflammatory bowel disease, allergies, and autoimmune diseases (De Filippis et al., 2021; Sultan et al., 2021; Christovich and Luo, 2022; Lee et al., 2022).

Intestinal commensal bacteria are recognized by pattern recognition receptors, including Toll-like receptors (TLRs), which act as sensors for microbes in the host. The intestinal epithelial cells (IECs) lining the intestinal mucosa separate the intestinal tract from the internal milieu. Thus, they are continuously exposed to commensal bacteria inhabiting the intestinal tract. IECs express some TLRs at much lower levels than other cell types such as monocytes (Abreu et al., 2001; Abreu et al., 2003; Melmed et al., 2003), reducing responsiveness to large quantities of commensal bacterial antigens, thereby preventing excessive inflammation. This indicates that the TLR expression in IECs is regulated through specific mechanisms different to those in other cell types. We previously reported that IECs express low levels of cell surface TLR4, which recognizes gram-negative bacteria, and respond minimally to its ligand, lipopolysaccharide (LPS), a potent inducer of inflammation. We further noted that decreased TLR4 expression in IECs is mediated by transcriptional suppression *via* DNA methylation (Takahashi et al., 2009). Interestingly, in the colon, which has a larger population of commensal bacteria compared to the small intestine, DNA methylation of the TLR4 gene in IECs depends on gut microbiota, indicating that commensal bacteria themselves maintain the symbiotic environment by causing epigenetic changes in host cells (Takahashi et al., 2011). In addition, we observed that the 5′ regions of specific populations of genes whose expression is downregulated by gut microbiota are highly methylated in colonic epithelial cells, suggesting that induction of DNA methylation serves as a mechanism by which the gut microbiota can regulate gene expression in IECs (Takahashi et al., 2020). However, the precise underlying molecular mechanisms remain to be elucidated.

DNA methylation is an epigenetic mechanism that controls gene expression. Usually, methyl groups are transferred to cytosine bases of CpG motifs on the target DNA, resulting in transcriptional repression *via* inactive chromatin status. In mammals, methyl group transfer is catalyzed by the methyltransferases DNMT1 and DNMT3. Of these, DNMT1 serves as a maintenance methyltransferase which predominantly methylates hemimethylated CpG nucleotides when DNA is replicated during cell division. In contrast, DNMT3 catalyzes *de novo* DNA methylation. IECs differentiate from the stem cells present in the crypt and move to the top of the villus during differentiation. Our previous observation showed that TLR4 gene methylation is more advanced in differentiated IECs than in undifferentiated IECs (Takahashi et al., 2011), indicating that *de novo* methylation of the TLR4 gene occurs during the differentiation process in IECs. Since DNMT3 mediates the transfer of methyl groups to any gene undergoing *de novo* methylation, the question of how gut microbiota induce the recruitment of DNMT3 to specific genes, including TLR4, remains to be addressed. In this study, we identified an adaptor molecule that recruits DNMT3b to the TLR4 gene and is induced by gut microbiota in colonic epithelial cells. This suggests a novel mechanism underlying epigenetic regulation of specific gene expression by gut microbiota in IECs.

## 2 Materials and methods

### 2.1 Cell culture

Human IEC lines Caco-2 (derived from epithelial colonic adenocarcinoma) and HCT116 (derived from epithelial colonic carcinoma) were purchased from DS Pharma Biomedical and cultured in Eagle’s MEM supplemented with nonessential amino acids and McCoy’s 5a medium, respectively. Another human IEC line SW480 (derived from epithelial colonic adenocarcinoma) was provided by the Cell Resource Center for Biomedical Research Institute of Development, Aging, and Cancer Center at Tohoku University (Miyagi, Japan) and cultured in a 1 : 1 mixture of L15 and DMEM. All media were supplemented with 10% (v/v) FBS, 100 U/mL of penicillin, 100 μg/ml streptomycin, and 5 × 10^–5^ M 2-mercaptoethanol. Cells were cultured at 37 °C in a humidified incubator with 5% CO_2_.

### 2.2 Methylation PCR

Genomic DNA was prepared from cells using a PureLink Genomic DNA Mini Kit (Invitrogen, Thermo Fisher Scientific, Waltham, MA, United States) and then subjected to bisulfite reactions using the EpiMark Bisulfite Conversion Kit (New England Biolabs, Ipswich, MA, United States) according to the manufacturer’s instructions. Quantitative PCR was performed with a LightCycler 480 (Roche, Basel, Switzerland), using the obtained DNA as a template. Combinations of the forward M and reverse, or forward U and reverse primers were used to amplify the region nt +98/+187 of the TLR4 gene, including methylated and unmethylated CpG motifs at nt +115, respectively (nucleotide numbers are counted from the major transcription start site as +1). The nucleotide sequence of each primer is shown below.

Forward M: 5′-GTG​TTT​TAG​AAA​TTG​TTC​GG-3′
Forward U: 5′-GTG​TTT​TAG​AAA​TTG​TTT​GG-3′
Reverse: 5′-TAA​CAT​CAT​CCT​CAC​TAC-3′

The methylation rate of the CpG motif at nt +115 was calculated by the following formula.

$$Methylation\;rate\left(\%\right)=1/\left(1+2^{-\left(Ct\left(u\right)-Ct\left(m\right)\right)}\right)\times 100$$

Ct(m), Ct value of PCR using forward M primer; Ct(u), Ct value of PCR using forward U primer.

### 2.3 Quantitative reverse transcription PCR

RNA was prepared from cells using the High Pure RNA isolation kit (Roche) followed by cDNA synthesis using SuperScript III or IV reverse transcriptase (Invitrogen, Thermo Fisher Scientific) and oligo (dT)_18_ primers (Invitrogen, Thermo Fisher Scientific). The mRNA expression levels of DNMT3a, DNMT3b, TLR4, RNA-binding motif protein (RBM) 14, IL-8, and GAPDH were quantified by real-time PCR using SYBR Green I Master reagent (Roche) or KAPA SYBR Fast qPCR kit (NIPPON Genetics, Tokyo, Japan) on a LightCycler 480 system (Roche) or CFX Connect real-time PCR detection system (Bio-Rad, Hercules, CA, United States). The following oligonucleotide pairs (F, forward; R, reverse) were used as PCR primers:

DNMT3a F: 5′-GAC​AAG​AAT​GCC​ACC​AAA​GC-3′
DNMT3a R: 5′-CGT​CTC​CGA​ACC​ACA​TGA​C-3′
DNMT3b F: 5′-AGC​TCT​TAC​CTT​ACC​ATC-3′
DNMT3b R: 5′-CCA​TCC​TGA​TAC​TCT​GAA-3′
TLR4 F: 5′-AAG​CCG​AAA​GGT​GAT​TGT​TG-3′
TLR4 R: 5′-CTG​AGC​AGG​GTC​TTC​TCC​AC-3′
RBM14 F: 5′-CCT​ACG​GCA​CGG​TCA​TGA​G-3′
RBM14 R: 5′-CGA​CAC​ATT​GCC​CAC​GAA​AA-3′
IL-8 F: 5′-GTG​CAG​TTT​TGC​CAA​GGA​GT-3′
IL-8 R: 5′-CTC​TGC​ACC​CAG​TTT​TCC​TT-3′
GAPDH F: 5′-TGA​ACG​GGA​AGC​TCA​CTG​G-3′
GAPDH R: 5′-TCC​ACC​ACC​CTG​TTG​CTG​TA-3′

The relative expression levels of *Dnmt3a*, *Dnmt3b*, *TLR4*, *RBM14*, and *IL-8* were calculated by normalizing to *Gapdh*.

### 2.4 Nuclear extract preparation

Cells were washed with PBS, resuspended in hypotonic buffer [10 mM HEPES, pH 7.9 containing 10 mM KCl, 0.1 mM EDTA, 1 mM DTT, and protease inhibitor cocktail (Nacalai Tesque, Kyoto, Japan)], and incubated on ice for 10 min. Cells were then treated with NP-40 at a concentration of 0.5% (v/v) and incubated on ice for an additional 15 min. After centrifuging at 4°C and 6,000 × g for 1 min, the surface of the pellet was washed with hypotonic buffer. Hypertonic buffer [10 mM HEPES, pH 7.9 containing 400 mM KCl, 4.5 mM MgCl_2_, 0.2 mM EDTA, 1 mM DTT, and protease inhibitor cocktail (Nacalai Tesque)] was then added followed by incubation on ice for 1 h. The supernatant obtained by centrifugation at 4°C, 10,000 × g, for 10 min was collected and used for experiments after the addition of 10% (v/v) glycerol. Protein concentration of the nuclear extract was determined using a Pierce^TM^ BCA Protein Assay Kit (Thermo Fisher Scientific).

### 2.5 Immunoprecipitation

Nuclear extracts containing 200 µg of protein were reacted with anti-DNMT3a (64B1446, Novus Biologicals, Centennial, CO, United States) or anti-DNMT3b (52A1018, Novus Biologicals) antibody in the presence of 0.1% NP-40 and protease inhibitor cocktail (Nacalai Tesque) at 4°C overnight with rotation. Dynabeads Protein G (Veritas, Thermo Fisher Scientific) were suspended in TNEN buffer (20 mM Tris-HCl, pH7.5 containing 150 mM NaCl, 1 mM EDTA, and 0.1% (v/v) NP-40), added to above mixture, and incubated at 4°C for 1 h with rotation. After washing the beads with TNEN buffer five times, proteins were eluted by incubation in SDS-PAGE sample buffer (50 mM Tris-HCl, pH 6.8 containing 20 mg/ml SDS, 10% (v/v) glycerol, 0.1 M DTT, and 1 mg/ml bromophenol blue) at 90°C for 5 min.

### 2.6 LC-MS/MS analysis

Immunoprecipitated proteins were separated by sodium dodecyl sulfate polyacrylamide gel electrophoresis and silver-stained using Pierce^TM^ Silver Stain for Mass Spectrometry (Thermo Fisher Scientific). The stained bands were excised from the gel, destained with Pierce^TM^ Silver Stain for Mass Spectrometry (Thermo Fisher Scientific), and reduced and alkylated, followed by tryptic digestion using an In-Gel Tryptic Digestion Kit (Thermo Fisher Scientific). After drying using a centrifugal evaporator, the product was dissolved in 0.1% (v/v) formic acid for LC-MS/MS analysis using Advance LC (AMR, Tokyo, Japan) and Q Exactive (Thermo Fishser Scientific). The data obtained were analyzed using Proteome Discoverer 2.1 software (Thermo Fisher Scientific) to search against the NCBI database using Sequent HT (Thermo Fishser Scientific). The mass spectrometry proteomics data have been deposited to the ProteomeXchange Consortium *via* the PRIDE (Perez-Riverol et al., 2022) partner repository with the dataset identifier PXD035777.

### 2.7 Chromatin immunoprecipitation assay

Cells were incubated in the presence of 1% (v/v) formaldehyde at 37°C for 10 min for crosslinking. After washing with PBS, cells were lysed with SDS lysis buffer [50 mM Tris-HCl, pH 8.1 containing 1% (w/v) SDS, 10 mM EDTA, and protease inhibitor cocktail (Nacalai Tesque)] and then sonicated using Vibra-Cell Processor (Sonics & Materials, Newtown, CT, United States) to shear DNA. ChIP was performed using a Chromatin Immunoprecipitation Assay Kit (Merck Millipore, Burlington, MA, United States). After dilution with ChIP dilution buffer supplemented with protease inhibitor cocktail (Nacalai Tesque), the lysates were incubated with protein A agarose/salmon sperm DNA at 4°C for 30 min with rotation for pre-clearing and centrifuged to collect the supernatant. The supernatant was dispensed into aliquots and incubated with antibodies against RBM14 (N1N2, GeneTex, Irvine, CA, United States), heterogeneous nuclear ribonucleoprotein (hnRNP) M1-M4 (1D8, Novus Biologicals), heat shock protein (HSP) 70 (3A3, StressMarq Biosciences, Victoria, Canada), HSP 90 (Merck Millipore) respectively, at 4°C overnight with rotation. Alternatively, the supernatant was reacted with ant-DNMT3b antibody (52A1018, Novus Biologicals) in the same manner. Normal mouse or rabbit IgG (Santa Cruz Biotechnology, Dallas, TX, United States; DA1E, Cell Signaling Technology, Danvers, MA, United States) was used as control. Protein A agarose/salmon sperm DNA was added, followed by incubation at 4°C for 1 h with rotation. After washing once with low salt immune complex wash buffer, once with high salt immune complex wash buffer, once with LiCl immune complex wash buffer, and twice with TE buffer, precipitates were eluted from the antibody in elution buffer (1% (w/v) SDS, 0.1 M NaHCO_3_) at room temperature for 15 min with rotation. The supernatant was collected *via* centrifugation. The elution process was repeated and the eluates were combined. The eluate was heated at 65°C for 4 h in the presence of 0.2 M NaCl to reverse crosslinks and then treated with proteinase K in the presence of 10 mM EDTA and 40 mM Tris-HCl pH6.5 at 45°C for 1 h. DNA was recovered by phenol/chloroform extraction, followed by ethanol precipitation. The obtained DNA was used as template for qPCR to amplify DNA regions of the TLR4 gene (nt −6439/−6301, −5272/−5121, −4275/−4154, −3021/−2844, −1939/−1760, −739/−628, +75/+201, +10224/+10408, +11243/+11417). Real-time PCR was performed using SYBR Green I master reagent (Roche) or KAPA SYBR Fast qPCR kit (NIPPON Genetics) on a LightCycler 480 system (Roche) or CFX Connect real-time PCR detection system (Bio-Rad). The DNA sequences of the primers used for qPCR are listed in Table 1.

**TABLE 1:** Sequences of primers used for ChIP assay.

Region		Sequence
−6439/−6301	F	5′-TTC​TGC​CCA​GAA​CAG​TGC​CTG-3′
	R	5′-GGG​TAA​ATG​AGA​TCT​TGT​GTG​TGG-3′
−5272/−5121	F	5′-GTG​AGC​ATC​ATA​CCA​GGC​CTC-3′
	R	5′-CCT​ACA​TAG​TGC​CAT​CTC​CTC​C-3
−4275/−4154	F	5′-GTA​GGG​GGA​AGA​GCC​TTG​AAG-3′
	R	5′-CCT​CTG​CTC​AGA​AGT​GAC​ATG​C-3
−3021/−2844	F	5′-TAT​GTG​ATG​GGG​GTA​GGC​CAG-3′
	R	5′-GCC​TCC​CAA​AGT​CTA​GAT​TCA​CC-3′
−1939/−1760	F	5′-TGC​CCA​GTC​CAC​CAC​AAA​ATG​G-3′
	R	5′-GGA​CAG​TGT​CTG​GAA​AGT​AGC​AAG-3′
−739/−628	F	5′-GCT​TAG​CGG​TTT​ACA​TGA​CTT​GAC​C-3′
	R	5′-CAA​GAA​GAT​TGG​GAA​AAG​TCT​GGG-3′
+75/+201	F	5′-GGC​TCG​AGG​AAG​AGA​AGA​CAC-3′
	R	5′-GGC​AGA​CAT​CAT​CCT​GGC​ATC-3′
+10224/+10408	F	5′-TTA​CCT​GGA​GTG​GGA​GGA​CAG-3′
	R	5′-CAG​CTG​GGC​AAG​AAA​TGC​CTC-3′
+11243/+11417	F	5′-CGC​TTC​CTG​GTC​TTA​TCA​TGG-3′
	R	5′-CCA​TCA​AAC​ATC​ACT​TGT​TCT​G-3′

### 2.8 RNA interference

Silencer Select siRNA of RBM14 or negative control (Ambion, Thermo Fisher Scientific) was introduced into HCT116 cells using X-tremeGENE HP (Roche) or Lipofectamine RNAiMAX (Invitrogen, Thermo Fisher Scientific) transfection reagents. After culturing for 72 h, total RNA and genomic DNA were isolated from the cells and analyzed by Quantitative reverse transcription PCR (qRT-PCR) and bisulfite sequencing, respectively. Alternatively, cells were subjected to the ChIP assay as described above.

### 2.9 LPS stimulation

HCT116 cells were stimulated with ultra-pure *Escherichia coli* K12 LPS (InvivoGen, San Diego, CA, United States) at concentrations of 0, 1, 10, 100 ng/ml 72 h after the introduction of siRNA. After culturing for an additional 16 h, induction of IL-8 gene expression was analyzed by qRT-PCR.

### 2.10 Bisulfite sequencing

EpiMark bisulfite conversion kit (New England BioLabs) was used for the bisulfite reaction of genomic DNA prepared from cells using a PureLink Genomic DNA Mini Kit (Invitrogen, Thermo Fisher Scientific). The nt −146/+461 region of the 5′ region of the TLR4 gene was amplified by PCR from the modified DNA. The nucleotide sequences of the primers used for PCR were as follows: 5′-GGT​AGA​GGT​TAG​ATG​ATT​AAT​TGG​G-3′ (forward) and 5′-CCC​CAA​TAA​CTA​CCT​CTA​ATA​CCC​T-3′ (reverse). PCR products were cloned into the pCR2.1 vector (Invitrogen, Thermo Fisher Scientific) and sequences were analyzed.

### 2.11 Mice

BALB/c mice were purchased from CLEA Japan (Tokyo, Japan) and bred under conventional (CV) or germ-free (GF) conditions. Female mice were used at 8–11 weeks of age. All experiments were approved by the Nihon University Animal Care and Use Committee and conducted in accordance with their guidelines.

### 2.12 IEC preparation

IECs of the medial small intestine and colon were prepared from mice as previously described (Sugi et al., 2016). In brief, 2–3 mm pieces of intestinal tissue sections were washed by shaking in HBSS supplemented with 1 mM DTT and 0.5 mM EDTA, followed by treatment with dispase (BD Biosciences, Franklin Lakes, NJ, United States). Lymphocytes were depleted from the single-cell suspensions using Dynabeads M-450 Streptavidin (Invitrogen, Thermo Fisher Scientific) and biotin-conjugated anti-CD45 antibody (eBioscience, San Diego, CA, United States).

### 2.13 Western blotting

Nuclear and cytoplasmic fractions were prepared from IEC lines using a Minute^TM^ cytoplasmic and nuclear extraction kit (Invent Biotechnology, Plymouth, MN, United States). Alternatively, whole cell lysates were prepared from human IEC lines or murine primary IECs as follows. Cells were washed with ice-cold PBS and incubated on ice for 30 min in lysis buffer (20 mM Tris pH 7.5, 150 mM NaCl, 1 mM EDTA, 60 mM n-octyl-β-D-glucoside, 1% Nonidet P-40) or RIPA buffer (50 mM Tris pH 7.6, 150 mM NaCl, 1% Nonidet P-40, 0.5% sodium deoxycholate, 0.1% SDS), both supplemented with protease inhibitor cocktail (Nacalai Tesque). After centrifugation at 4 °C and 20, 000 × g for 10 min, supernatants were collected. The obtained nuclear, cytoplasmic, or whole cell lysates were subjected to western blotting using a combination of anti-RBM14 Ab (GTX112293, GeneTex, Irvine, CA, United States; ab70636, Abcam) as the primary antibody and HRP-linked anti-rabbit IgG Ab (Cell Signaling Technology) as the secondary Ab, or HRP-linked anti-β-actin Ab (ab49900, Abcam). After chemiluminescent detection, band intensities were calculated using ImageJ software.

### 2.14 Statistics

Differences between two or more groups were analyzed using two-tailed Student’s *t*-test or one-way ANOVA followed by Tukey’s test, respectively. Results with *p*-value <0.05 were considered statistically significant.

## 3 Results

### 3.1 TLR4 expression in IECs depends on DNA methylation but not on DNA methyltransferase expression levels

**FIGURE 1:**
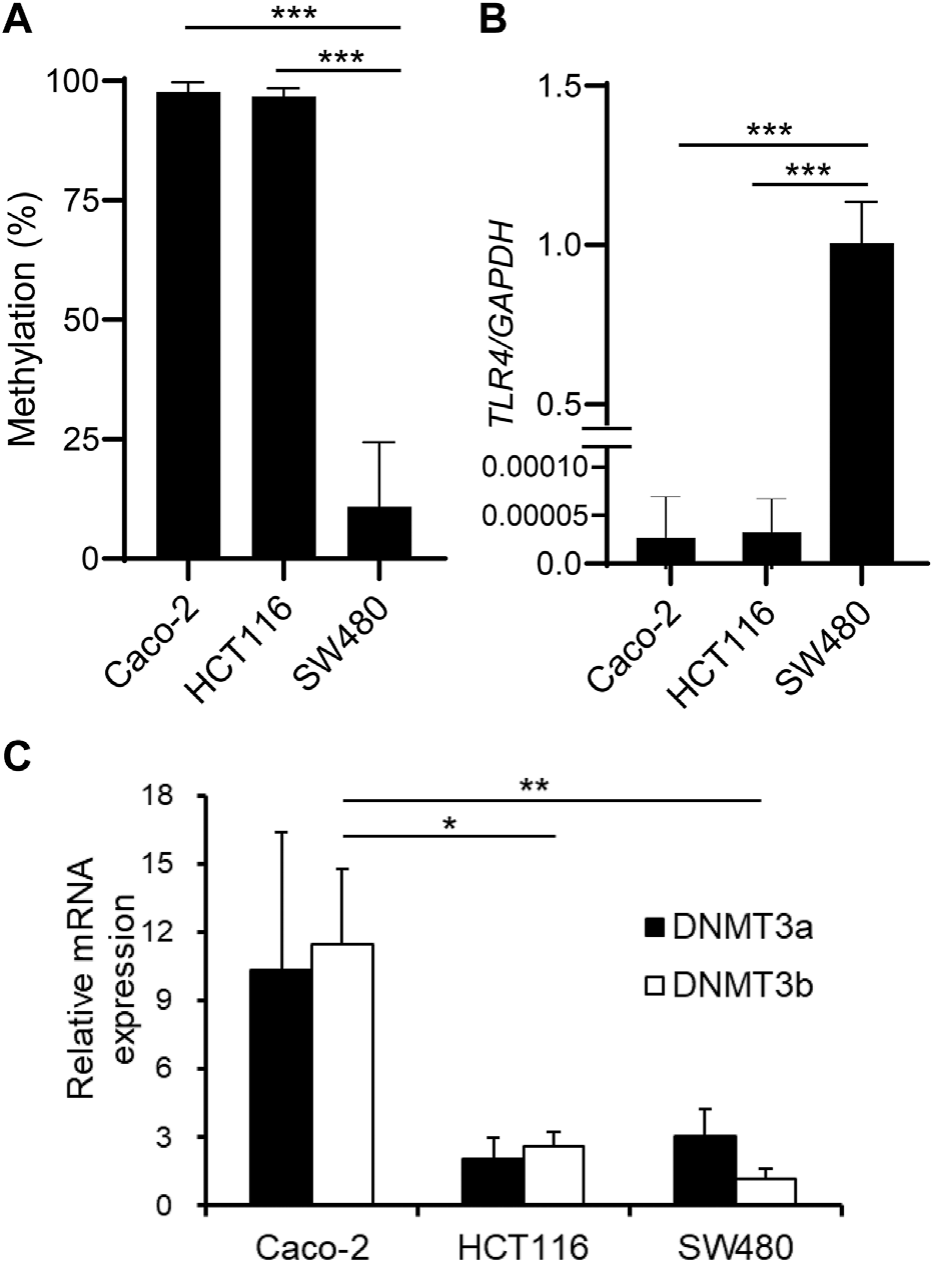
DNA methylation of the TLR4 gene does not correlate with DNMT expression levels in IEC lines. **(A)** DNA methylation status of the CpG motif at nt +115 of the TLR4 gene in human IEC lines Caco-2, HCT116, and SW480 was determined by methylation PCR. Results are expressed as methylation percentage of this motif and shown as mean ± SD of three independent experiments. *** Significantly different (*p* < 0.0001). **(B)** mRNA expression of TLR4 in Caco-2, HCT116, and SW480 cells was analyzed by qRT-PCR. Relative expression levels are shown as mean ± SD of three independent experiments. *** Significantly different (*p* < 0.0001). **(C)** mRNA expression of DNMT3a (filled bars) and DNMT3b (open bars) in Caco-2, HCT116, and SW480 cells was analyzed by qRT-PCR. Results are shown as mean ± SD of three independent experiments. *, ** Significantly different (*p < 0.05, **p < 0.01).

We have previously reported that TLR4 gene expression is downregulated in IECs through DNA methylation. It was shown that IECs exhibit higher DNA methylation and lower expression of the TLR4 gene compared to other cell types *in vivo* using IECs and splenocytes from mice (Takahashi et al., 2011). Human IEC lines such as Caco-2 and HCT116 also display hypermethylation and low expression of the TLR4 gene, while another line SW480 shows hypomethylation and high expression of the TLR4 gene (Takahashi et al., 2009). First, these three IEC lines were compared for CpG methylation status in the 5′ region of the TLR4 gene by methylation PCR with primers constructed to include the CpG motif at nt +115 (nucleotide numbers are counted from the major transcription start site as +1). Similar to the results obtained by bisulfite sequence analysis covering the region from nt -69 to nt +277 in the previous report (motif no. 5 corresponds to the CpG motif at nt +115), DNA methylation frequency of the 5′ region of the TLR4 gene was shown to be significantly higher in Caco-2 and HCT116 cells than in SW480 cells (Figure 1A). TLR4 mRNA expression was also confirmed to be extremely low in Caco-2 and HCT116 cells compared to that in SW480 cells (Figure 1B). In contrast, mRNA expression of DNMT3b was significantly higher in Caco-2 cells than in HCT116 and SW480 cells (Figure 1C). DNMT3a expression was also higher in Caco-2 cells than in HCT116 and SW480 cells, although the difference was not statistically significant. As no correlation was observed between DNA methylation and DNMT3 expression levels, it was proposed that the hypermethylation of the TLR4 gene was not due to increased DNMT3 expression. In particular, DNMT3 was expressed at similar levels in HCT116 and SW480 cells, which presented hypermethylation and hypomethylation of the TLR4 gene, respectively, suggesting that comparison of molecules that interact with DNMT3 between these cell lines would allow for the identification of factors involved in the recruitment of DNMT3 to the TLR4 gene.

### 3.2 RBM14 was identified as a DNMT3-interacting protein specific to the IEC line with hypermethylated TLR4 gene

**FIGURE 2:**
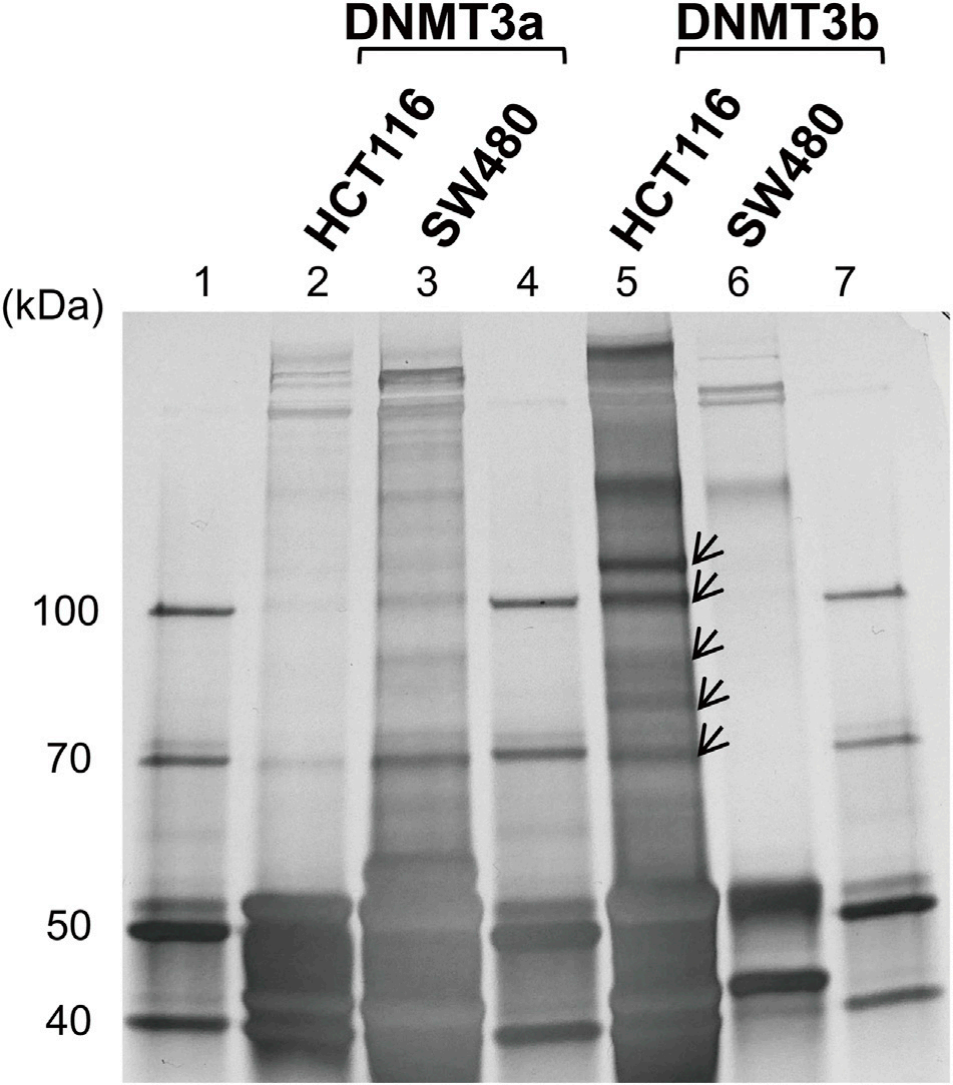
Identification of DNMT-interacting nuclear factors specific to the IEC line with hypermethylated TLR4 gene. Pull-down assays of the nuclear extracts from HCT116 and SW480 cells were performed using antibodies against DNMT3a and DMNT3b. The precipitates were analyzed by silver staining. Lanes 1, 4, and 7, molecular-weight size marker; lane 2, HCT116 nuclear extract immunoprecipitated with anti-DNMT3a antibody; lane 3, SW480 nuclear extract immunoprecipitated with anti-DNMT3a antibody; lanes 5, HCT116 nuclear extract immunoprecipitated with anti-DNMT3b antibody; lane 6, SW480 nuclear extract immunoprecipitated with anti-DNMT3b antibody. Arrows indicate bands specific to the nuclear extract of HCT116 cells and are not or minimally observed in that of SW480 cells. Representative image of three independent experiments is shown.

To identify DNMT3-interacting molecules specifically present in the nuclear extracts of HCT116 cells rather than those of SW480 cells, pull-down assays were performed using antibodies against DNMT3a and DNMT3b (Figure 2). Analysis of the precipitates by silver staining resulted in the appearance of bands specific to HCT116, as indicated by arrows when anti-DNMT3b antibody was used. No bands specific to HCT116 cells were observed when DNMT3a antibody was used for the assay. Specific bands were subjected to in-gel trypsin digestion followed by MS analysis. Among the proteins identified, those with nucleic acid binding activity were further selected using the Gene Ontology Consortium (http://www.geneontology.org), yielding four candidate molecules (Table 2): RBM 14, hnRNP M, HSP 70, and HSP 90. These proteins were considered to interact with DNMT3b preferentially in HCT116 cells with hypermehtylated TLR4 gene compared to SW480 cells with hypomethylated TLR4 gene.

**TABLE 2:** Candidate proteins with DNA binding ability. Numbers of peptide spectrum matches (PSMs), peptides, and unique peptides not present in other proteins, and percentages of coverage are shown.

Protein name	NCBI accession no.	PSMs	Peptides	Unique peptides	Coverage (%)
HSP 90	AAA36025	4	4	1	5.2
hnRNP M	AAH19580	11	7	2	12.3
RBM 14	KAI4072531	12	7	7	13.8
HSP 70 protein 8	AAH19816	18	10	8	20.4

### 3.3 RBM14 binds to the TLR4 gene in the IEC line with hypermethylated TLR4 gene

**FIGURE 3:**
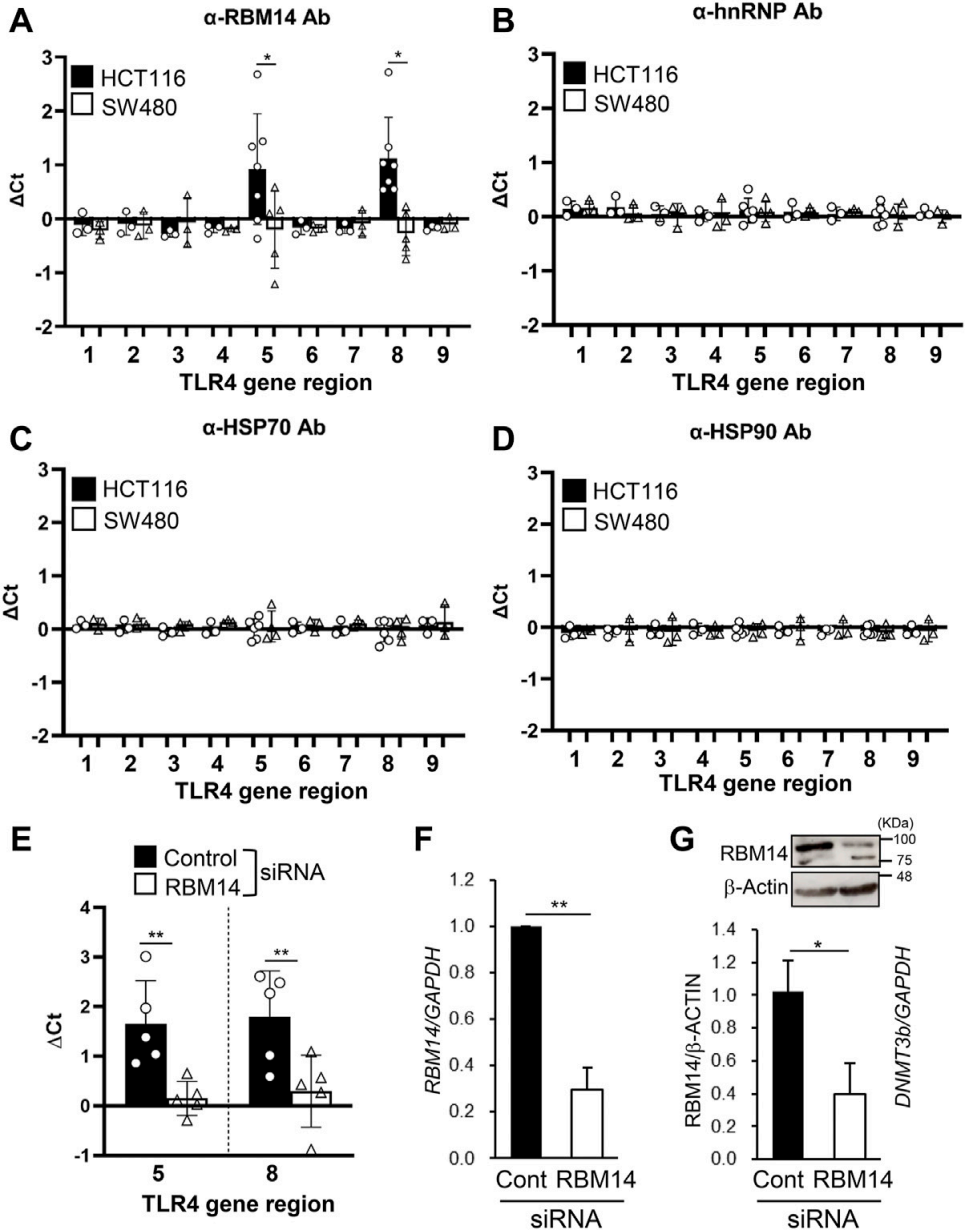
RBM14 binds to the TLR4 gene in the IEC line with hypermethylated TLR4 gene. **(A–E)** ChIP assays of HCT116 and SW480 were performed using antibodies against RBM14 **(A)**, hnRNP **(B)**, HSP70 **(C)**, and HSP90 **(D)**. Anti-DNMT3b antibody was used for ChIP assays in HCT116 cells treated with control or RBM14 siRNA **(E)**. PCR primers were prepared to cover the 5′ regions, nt −6439/−6301 (region 1), −5272/−5121 (region 2), −4275/−4154 (region 3), −3021/−2844 (region 4), −1939/−1760 (region 5), −739/−628 (region 6), and +75/+201 (region 7), and the 3′ regions, nt +10224/+10408 (region 8) and +11243/+11417 (region 9), of the TLR4 gene. △Ct was calculated by subtracting the Ct value of qPCR analysis of the precipitates with each antibody from that with corresponding isotype control antibody. Results are shown as mean ± SD of 3-7 independent experiments. *, ** Significantly different (*p < 0.05, **p < 0.005). **(F)** RBM14 mRNA expression in HCT116 cells treated with negative control siRNA or RBM14 siRNA was determined using qRT-PCR. Relative expression levels to control cells are shown as mean ± SD of four independent experiments. ** Significantly different (*p* < 0.001). **(G)** Expression of RBM14 in control or RBM14 siRNA-treated HCT116 cells was analyzed using western blotting. Representative blots (top) and mean ± SD of relative band intensities normalized to those of β-actin of three independent experiments (bottom) are shown. * Significantly different (*p* < 0.05).

Next, we tested whether these proteins interacted with the DNA regions of the TLR4 gene by ChIP assays with antibodies against each protein. PCR primers were prepared to cover the 5´ (nt −6439 to nt +201) and the 3´ (nt +10224 to nt +11417) regions of the TLR4 gene, as shown in Table 1. Significant amplification of PCR products corresponding to the regions nt −1939/−1760 and nt +10224/+10408 was detected when using the RBM14 antibody compared to the isotype control antibody (*p* < 0.05, Figure 3A). In contrast, no PCR products were amplified when using anti-hnRNP M, anti-HSP 70 and anti-HSP 90 antibodies (Figures 3B–D). These results revealed that RBM14, identified as a factor binding to DNMT3b in Figure 2, interacts with specific DNA regions of the TLR4 gene. As RBM14 was shown to physically interact with both DNMT3b and the DNA regions of the TLR4 gene, it indicates that RBM14 can act as an adaptor to recruit DNMT3b to the TLR4 gene. Actually, DNMT3b was shown to interact with these regions in ChIP assays using anti-DNMT3b antibody (Figure 3E). In addition, RBM14 siRNA was introduced into HCT116 cells. The decrease in RBM14 mRNA and protein expression induced by siRNA was confirmed by qRT-PCR and western blotting, respectively (Figures 3F,G). Knockdown of RBM14 reduced the interaction of DNMT3b with the nt −1939/−1760 and nt +10224/+10408 regions, indicating that DNMT3b is recruited to these DNA regions in an RBM14-dependent manner (Figure 3E).

### 3.4 RBM14 induces DNA methylation of the TLR4 gene

**FIGURE 4:**
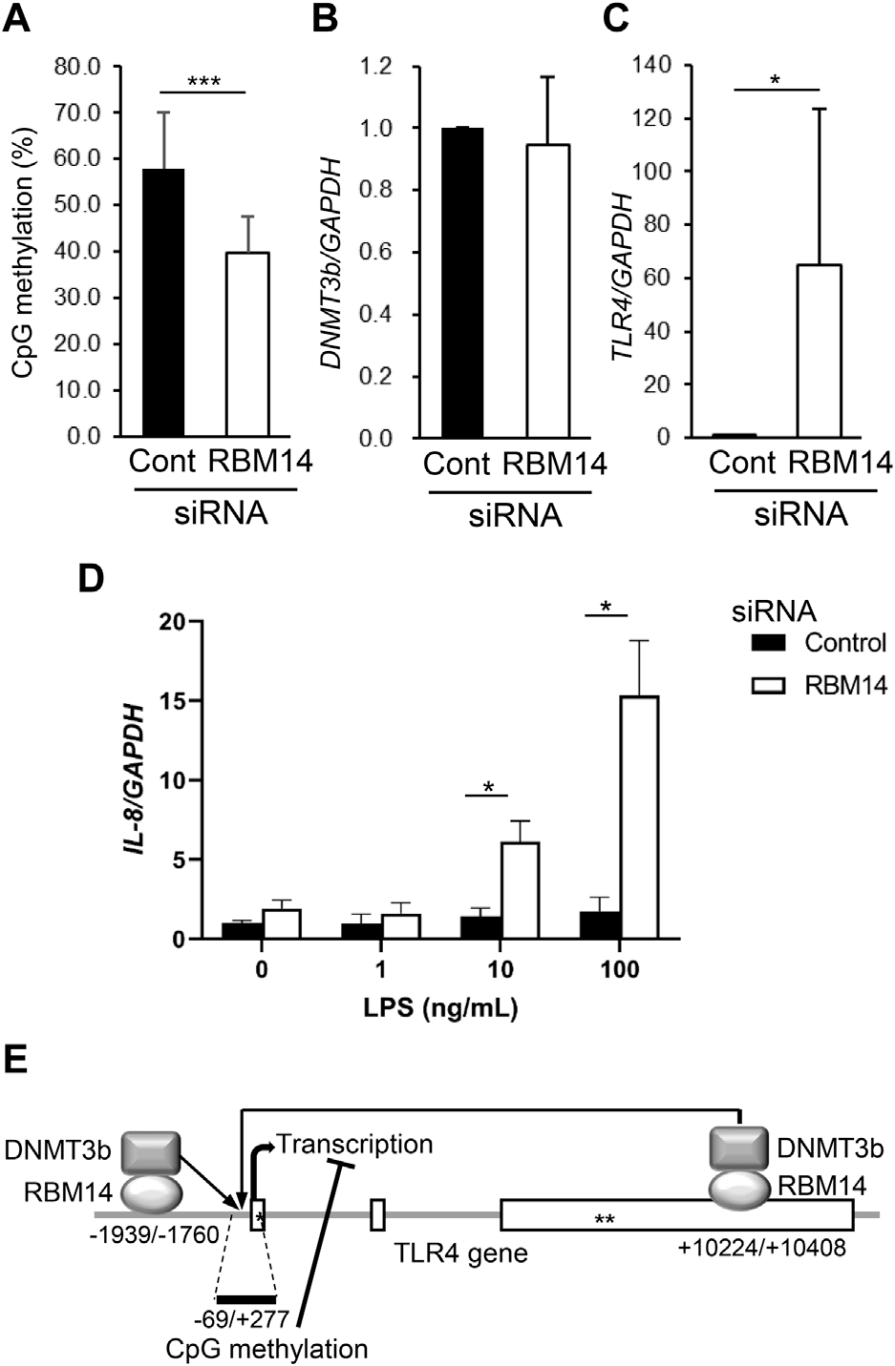
RBM14 induces DNA methylation of the TLR4 gene. RBM14 siRNA (open bars) or negative control siRNA (filled bars) was introduced into HCT116 cells. **(A)** Methylation frequency of CpG motifs present in the nt -69/+277 region of the TLR4 gene was analyzed by bisulfite sequencing. Sequences of total 17 (RBM14 siRNA) and 18 (negative control siRNA) clones obtained from three independent experiments were analyzed. *** Significantly different (*p* < 0.0001). **(B)** DNMT3b mRNA expression was determined by qRT-PCR. Relative expression levels to control cells are shown as mean ± SD of three independent experiments. **(C)** TLR4 mRNA expression was determined by qRT-PCR. Relative expression levels to control cells are shown as mean ± SD of three independent experiments. * Significantly different (*p* < 0.05). **(D)** Induction of IL-8 mRNA expression upon LPS stimulation was analyzed by qRT-PCR. Results are shown as mean ± SD of three independent experiments. * Significantly different (*p* < 0.05). **(E)** Schematic diagram of the interaction of RBM14 with the TLR4 gene and DNMT3b underlying the induction of TLR4 gene methylation. Open boxes and asterisks (* and **) represent the exons, start codon, and stop codon, respectively.

To determine whether the recruitment of DNMT3b to the TLR4 gene by RBM14 leads to DNA methylation, methylation of CpG motifs in the region from nt -69 to nt +277 of the TLR4 gene was analyzed in RBM14 siRNA-treated HCT116 cells. Methylation frequency was significantly decreased in cells treated with RBM14 siRNA compared to those treated with control siRNA (Figure 4A), indicating that recruitment of DNMT3b to the TLR4 gene by RBM14 results in DNA methylation. In contrast, DNMT3b mRNA expression was not affected by knockdown of RBM14 (Figure 4B). Knockdown of RBM14 significantly increased TLR4 gene expression (Figure 4C), suggesting that it cancelled the methylation-mediated transcriptional repression of the TLR4 gene. Furthermore, induction of IL-8 expression upon LPS stimulation was not observed in control cells, but was significantly increased by RBM14 knockdown (Figure 4D). A schematic diagram of the interaction of RBM14 with the TLR4 gene and DNMT3b, which induces DNA methylation of the TLR4 gene, is shown in Figure 4E.

### 3.5 RBM14 is upregulated by gut microbiota in colonic epithelial cells

**FIGURE 5:**
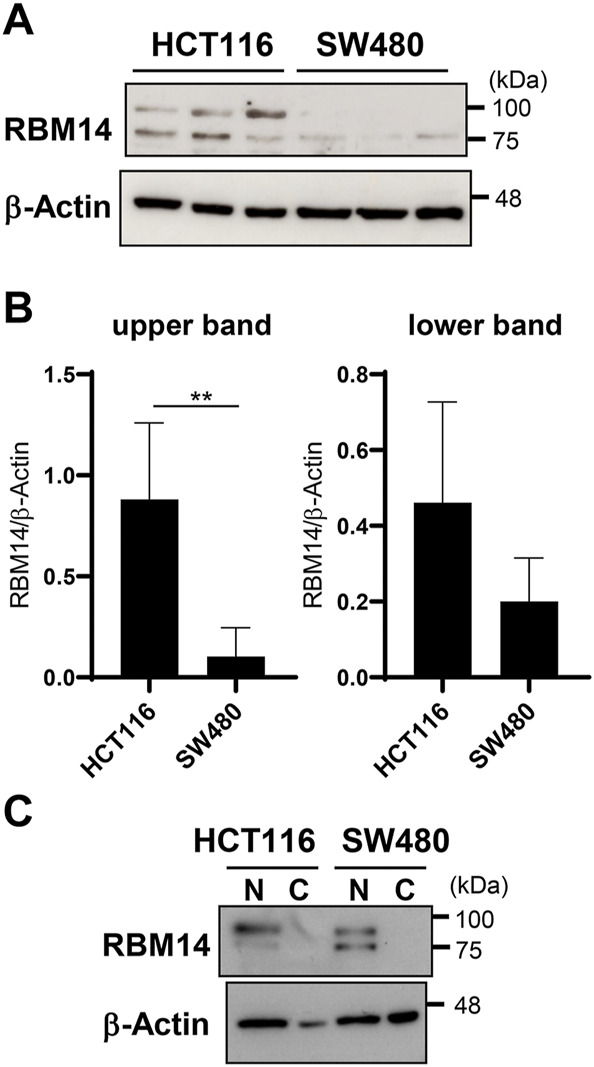
RBM14 is expressed at higher levels in the IEC line with hypermethylated TLR4 gene than in that with hypomethylated TLR4 gene. **(A** and **B)** Expression of RBM14 in HCT 116 and SW480 cells was examined by western blotting using whole cell lysates. Lysates prepared from five independent cultures for each cell line was analyzed. Representative blots of each three independent lysates are shown in **(A)**. Relative band intensities normalized to those of β-actin were calculated for the upper and lower band of RBM14, respectively **(B)**. Results are expressed as mean ± SD of five independent experiments. ** Significantly different (*p* < 0.005). **(C)** Nuclear and cytoplasmic fractions of cell lysates were separately prepared from HCT116 and SW480 cells and the expression of RBM14 was analyzed by western blotting. Representative blots of two independent experiments are shown.

Next, RBM14 expression in HCT116 and SW480 cells was analyzed by western blotting (Figures 5A,B). RBM14 appeared as double bands, the lower of which had a molecular mass close to the calculated molecular mass of approximately 70 kDa, suggesting that the other upper band was caused by additional modifications. Both forms of RBM14 were expressed more abundantly in HCT116 cells than in SW480 cells, indicating that the expression of RBM14 was higher in cells with hypermethylated TLR4 gene than in those with hypomethylated TLR4 gene. In particularly, the higher molecular mass form was significantly more abundant in HCT116 cells than in SW480 cells (*p* < 0.005). No significant difference in the expression of lower molecular mass form was identified. When cell lysates of the nuclear and cytoplasmic fractions were separately prepared, RBM14 was only detected in the nuclear fractions, indicating that RBM14 was mainly present in the nucleus (Figure 5C). The higher molecular mass form was also more abundant in the nuclear fractions of HCT116 cells than in those of SW480 cells, but no such trend was observed for the lower molecular mass form.

**FIGURE 6:**
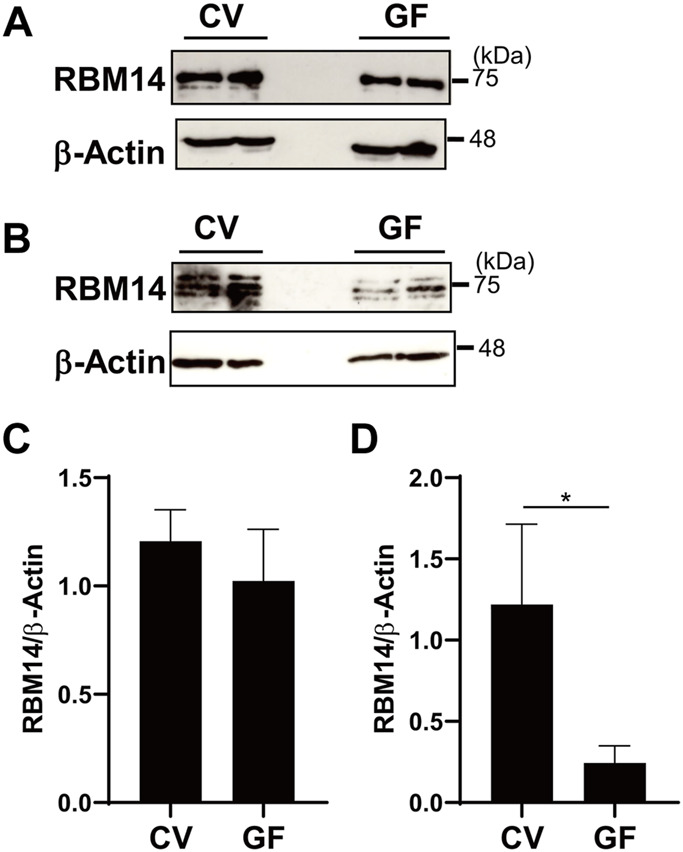
Expression of RBM14 is induced by gut microbiota in colonic epithelial cells. Expression of RBM14 in epithelial cells from medial small intestine **(A** and **C)** and colon **(B** and **D)** of CV and GF mice was analyzed by western blotting. Representative blots **(A** and **B)** and mean ± SD of relative band intensities normalized to those of β-actin **(C** and **D)** of four independent experiments are shown. The band intensities was measured as total of all three bands because triplet bands did not always separate clearly. IECs pooled from 3-6 mice were used for each experiment. * Significantly different (*p* < 0.05).

In addition, *in vivo* expression of RBM14 was analyzed using epithelial cells prepared from the medial small intestine and colon of mice bred under CV and GF conditions. As shown in Figures 6A,B, RBM14 was expressed in IECs from both the small intestine and colon. Triplet bands appeared, of which the middle band was expressed at the highest level in both IECs from the small intestine and colon. In colonic epithelial cells, the expression of RBM14 was significantly higher in CV mice than in GF mice, indicating that RBM14 expression is induced by the gut microbiota (Figures 6B,D). Particularly, the bands with the highest molecular mass markedly increased in intensity in colonic epithelial cells of CV mice compared to those of GF mice. However, no significant difference in RBM14 expression was observed in small intestinal epithelial cells between CV and GF mice (Figures 6A,C).

Collectively, it was concluded that RBM14, whose expression is induced by gut microbiota, binds to the TLR4 gene and recruits DNMT3b to induce DNA methylation of the gene in colonic epithelial cells. These findings explain how gut microbiota epigenetically regulate specific gene expression through DNA methylation despite the use of common DNMT and identify a novel mechanism underlying gut microbiota-mediated maintenance of intestinal homeostasis.

## 4 Discussion

The impact of the gut microbiota on the host immune system and health has attracted considerable attention in recent years. However, the precise mechanisms underlying the prevention of immunological exclusion of the gut microbiota and excessive inflammatory reactions have not been fully elucidated. Decreased sensitivity to gut microbes by suppression of specific TLR expression in IECs is believed to play a key role. We previously reported that TLR4 gene expression is epigenetically suppressed by gut microbiota-dependent DNA methylation in colonic epithelial cells (Takahashi et al., 2011). However, the question remains as to how common DNMT induce methylation of specific genes, including TLR4. Here, we identified RBM14, which was expressed at higher levels in the nuclei of an IEC line with hypermethylated TLR4 gene than in that with hypomethylated TLR4 gene, as an adaptor molecule that can recruit DNMT3 to the TLR4 gene. A schematic diagram is shown in Figure 4E, but it remains unclear whether RBM14 binds directly or indirectly to DNMT3b and to the DNA regions of the TLR4 gene. Future studies are required to clarify which region or conformation of RBM14 mediates potential direct binding, and what molecules besides RBM14, DNMT3b, and DNA regions of the TLR4 gene constitute the complex in the case of indirect binding. Studies observing mutant RBM14 with amino acid substitutions that mediate its interaction with DNMT3b will be useful in elucidating the function of RBM14 in the control of the TLR4 gene.

In addition, expression of RBM14 was induced by gut microbiota in colonic epithelial cells but not in small intestinal epithelial cells (Figure 6), which is consistent with our previous data showing that DNA methylation of the TLR4 gene depends on gut microbiota in colonic epithelial cells but not in small intestinal epithelial cells (Takahashi et al., 2011). As more than 99% of the intestinal commensal bacteria inhabit the colon, this may indicate that gut microbiota-dependent regulation of inflammation is more critical in the colon than in the small intestine. In fact, there is probably a complicated regulatory mechanism for TLR4 gene expression in the intestinal epithelium. Price et al. generated reporter mice to visualize the expression of TLRs and observed higher expression of TLR4 on IECs in the colon than in the small intestine (Price et al., 2018), which is consistent with the mRNA expression pattern in our previous report. It is theorized that there is a mechanism independent of DNA methylation that upregulates the TLR4 gene in colonic epithelial cells, and that gut microbiota-dependent DNA methylation prevents overexpression of the TLR4 gene and excessive responses to microbes. Future studies are needed to confirm the effect of gut microbiota by conventionalization of GF mice and to identify bacteria and their components and/or metabolites with high activity in inducing RBM14 expression using a gnotobiotic mouse model. Although it is currently unclear what stimuli from commensal bacteria induce RBM14 expression, there may be a negative feedback system in that increased recognition of commensal bacteria upregulates RBM14 expression, which in turn suppresses TLR4 expression. Other stimuli, such as those derived from food components that are absorbed from the small intestine and rarely reach the colon, may induce RBM14 expression in small intestinal epithelial cells in a manner independent of the gut microbiota. Although the biological significance of RBM14 expression in the small intestine is currently unknown, it may be involved in intestinal homeostasis by regulating genes other than TLR4.

RBM14 was first identified as an activator of the general coactivator, thyroid hormone receptor-binding protein (TRBP), and is known as a coactivator activator (CoAA). TRBP enhances transcription mediated by nuclear receptors and other transcription factors, such as NF-κB and AP-1, *via* interaction with CBP/p300 histone acetyl transferases (HAT). As RBM14 was shown to interact with both TRBP and p300, it is considered to activate transcription by acting synergistically with TRBP and CBP/p300 (Iwasaki et al., 2001). RBM14 was also reported to suppress c-myc gene expression by recruiting histone deacetylase (HDAC) (Kang et al., 2008), indicating that it is involved in transcriptional regulation through interactions with histone-modifying enzymes such as HAT and HDAC. RBM14 has two RNA recognition motifs and has been shown to control RNA splicing coordinately with transcription (Auboeuf et al., 2002; Auboeuf et al., 2004). Moreover, it plays a role in DNA repair (Yuan et al., 2014) and maintenance of mitotic spindle integrity (Shiratsuchi et al., 2015). Based on these reports, it is possible that RBM14 directly and indirectly binds to DNA. In the latter case, RBM14 may bind to other DNA-binding nuclear or transcription factors, as observed in the case of Runx and PEA3 transcription factors (Li et al., 2009; Verreman et al., 2011), thereby indirectly interacting with DNA.

RBM14 was found to bind to the DNA regions nt −1939/−1760 and nt +10224/+10408 of the TLR4 gene by ChIP assays (Figure 3A), and DNA methylation in the region nt -69/+277 of the TLR4 gene was suppressed by the knockdown of RBM14 in the IEC line (Figure 4A), which led to an increase in TLR4 gene expression (Figure 4C). Although methylation of CpG motifs was observed in the 5′ region around the transcription start site, which appeared to be separated from these regions in the primary structure, as seen in Figure 4E, it was considered that these regions may become closer to the methylation sites in the high-dimensional chromatin structure. The inhibitory effect observed in Figure 4A seemed to be partial, likely due to the culture period after transfection. Analysis of *de novo* methylation of the TLR4 gene in cells of the new generation was necessary, but the effect of siRNA is not always sustained over cell generations, which limits culture period. Although methylation of CpG motifs present in regions other than nt –69/+277 was not analyzed in this study, this possibly occurs around nt −1939/−1760 and nt +10224/+10408 regions in addition to the region proximal to the transcription start site.

RBM14 protein was mainly expressed in the nucleus (Figure 5C), consistent with its role in DNA binding and recruitment of DNMT found in this study, as well as previously known roles such as CoAA, controlling RNA splicing, and DNA repair. RBM14 was detected by western blotting as doublet bands in human IEC lines, of which the higher molecular mass form was particularly abundant in IECs with the hypermethylated TLR4 gene (Figures 5A,B). The higher molecular mass form probably possesses some post-translational modifications such as glycosylation. Although the presence of a splicing variant with a lower molecular mass of RBM14 has been reported (Kang et al., 2008), post-translational modifications of RBM14 have not been analyzed. Post-translational modifications of proteins include various aspects such as phosphorylation, acetylation, and glycosylation, of which N-glycosylation causes a relatively large increase in the molecular mass. There are three and two N-glycosylation motifs (Asn-X-Ser/Thr) in human and mouse RBM14, respectively, two of which are conserved between humans and mice. We do not rule out the possibility that RBM14 contains modifications other than glycosylation, and how each modification affects the activity of RBM14 needs to be clarified. RBM14 was also detected in multiple molecular mass forms in murine primary IECs (Figure 6). The diverse band patterns observed suggest that post-translational modifications vary among organisms and may also vary depending on the cell type and activation state. The highest molecular mass form, shown to be markedly upregulated by the gut microbiota in colonic epithelial cells, may correspond to the higher molecular mass form detected in human IEC lines, although these slightly differed in molecular mass. The lowest molecular mass observed in murine primary IECs was not detected in human IEC lines. Therefore, it is considered that the modified, high molecular mass form of RBM14, significantly abundant in IECs with hypermethylated TLR4 gene and induced by gut microbiota, binds to DNMT3b and is involved in the recruitment of DNMT3b to the TLR4 gene.

Our findings support the idea that the gut microbiota induce DNA methylation of specific genes by upregulating adaptor molecules that recruit DNMT to target genes. Interestingly, hnRNP M, an hnRNP member, was shown to interact with DNMT3 (Table 2), although it did not interact with the DNA regions of the TLR4 gene (Figure 3B). As RBM14 is an hnRNP-like protein, it is possible that hnRNP family members play a role in inducing DNA methylation of specific genes by binding to different target genes to recruit DNMT3. Multiple classes of adaptors may bind to different gene populations and specifically recruit DNMT to corresponding gene population, which might explain why DNA methylation only occurs on certain genes despite the use of common DNMT enzymes. Furthermore, the expression of some of the adaptor molecules, including RBM14, appears to be regulated by the gut microbiota, suggesting that this is an underlying mechanism of the epigenetic regulation of specific genes in IECs by gut microbiota. As altered DNA methylation in IECs has been reported in diseases known to involve dysbiosis of the gut microbiota, such as colonic carcinoma and inflammatory bowel disease (Howell et al., 2018; Sobhani et al., 2019), the expression of some adaptor molecules may be affected under these conditions. Elucidation of the relationship between the composition of gut microbiota and RBM14 expression in IECs and identification of bacteria that induce RBM14 expression will be important for application of microbiota intervention approaches to control diseases involving intestinal inflammation.

RBM14 interacted only with specific regions, but not with many other regions, of the TLR4 gene (Figure 3A), suggesting that there is some nucleotide sequence specificity in its binding. It is speculated that RBM14 targets not only the TLR4 gene, but also a specific population, not all, of the genes whose methylation is induced by the gut microbiota. Future studies identifying other genes bound by RBM14 to induce DNA methylation will be helpful in understanding how the gut microbiota contributes to maintaining intestinal homeostasis through upregulation of RBM14. Analysis of the induction and/or modification of RBM14 by various stimuli is also necessary to clarify the importance of gut microbiota composition for this epigenetic regulation. In particular, it is expected to elucidate how such mechanisms contribute to the regulation of genes involved in inflammatory reactions as inflammation control plays a key role in maintenance of the intestinal symbiotic ecosystem. Previous studies, including ours, have shown that gut microbiota themselves prepare environments that prevent their immunological exclusion and intestinal inflammation (Maslowski et al., 2009; Round and Mazmanian, 2010; Atarashi et al., 2011; Furusawa et al., 2013; Sujino et al., 2016; Kuhn et al., 2018; Maerz et al., 2018; Erturk-Hasdemir et al., 2019; Martínez-López et al., 2019), suggesting the involvement of such mechanisms in this regulation. Collectively, the results obtained in this study indicate a novel function of RBM14 as an adaptor molecule that recruits DNMT3 to target genes and further our understanding of the mechanisms underlying the microbiota-dependent maintenance of intestinal homeostasis.

## Data Availability

The datasets presented in this study can be found in online repositories. The names of the repository/repositories and accession number(s) can be found below: http://proteomecentral.proteomexchange.org/cgi/GetDataset PXD035777.

## Abbreviations

TLR, Toll-like receptor; DNMT, DNA methyltraneferase; IEC, intestinal epithelial cell; LPS, lipopolysaccharide; ChIP, chromatin immunoprecipitation; RBM, RNA-binding motif protein; hnRNP, heterogeneous nuclear ribonucleoprotein; HSP, heat shock protein; CV, conventional; GF, germ-free; TRBP, thyroid hormone receptor-binding protein; CoAA, coactivator activator; HAT, histone acetyl transferase; HDAC, histone deacetylase.

## References

[B1] Abreu M. T. Thomas L. S. Arnold E. T. Lukasek K. Michelsen K. S. Arditi M. J. (2003). TLR signaling at the intestinal epithelial interface. J. Endotoxin Res. 9 (5), 322–330. 10.1179/096805103225002593 14577850

[B2] Abreu M. T. Vora P. Faure E. Thomas L. S. Arnold E. T. Arditi M. (2001). Decreased expression of Toll-like receptor-4 and MD-2 correlates with intestinal epithelial cell protection against dysregulated proinflammatory gene expression in response to bacterial lipopolysaccharide. J. Immunol. 167 (3), 1609–1616. 10.4049/jimmunol.167.3.1609 11466383

[B3] Atarashi K. Tanoue T. Shima T. Imaoka A. Kuwahara T. Momose Y. (2011). Induction of colonic regulatory T cells by indigenous Clostridium species. Science 331 (6015), 337–341. 10.1126/science.1198469 21205640 PMC3969237

[B4] Auboeuf D. Dowhan D. H. Li X. Larkin K. Ko L. Berget S. M. (2004). CoAA, a nuclear receptor coactivator protein at the interface of transcriptional coactivation and RNA splicing. Mol. Cell. Biol. 24 (1), 442–453. 10.1128/MCB.24.1.442-453.2004 14673176 PMC303353

[B5] Auboeuf D. Hönig A. Berget S. M. O'Malley B. W. (2002). Coordinate regulation of transcription and splicing by steroid receptor coregulators. Science 298 (5592), 416–419. 10.1126/science.1073734 12376702

[B6] Bostick J. W. Schonhoff A. M. Mazmanian S. K. (2022). Gut microbiome-mediated regulation of neuroinflammation. Curr. Opin. Immunol. 76, 102177. 10.1016/j.coi.2022.102177 35462279 PMC9167715

[B7] Brodin P. (2022). Immune-microbe interactions early in life: A determinant of health and disease long term. Science 376 (6596), 945–950. 10.1126/science.abk2189 35617387

[B8] Christovich A. Luo X. M. (2022). Gut microbiota, leaky gut, and autoimmune diseases. Front. Immunol. 13, 946248. 10.3389/fimmu.2022.946248 35833129 PMC9271567

[B9] Cook T. M. Mansuy-Aubert V. (2022). Communication between the gut microbiota and peripheral nervous system in health and chronic disease. Gut Microbes 14 (1), 2068365. 10.1080/19490976.2022.2068365 35482894 PMC9067538

[B10] De Filippis F. Paparo L. Nocerino R. Della Gatta G. Carucci L. Russo R. (2021). Specific gut microbiome signatures and the associated pro-inflamatory functions are linked to pediatric allergy and acquisition of immune tolerance. Nat. Commun. 12, 5958. 10.1038/s41467-021-26266-z 34645820 PMC8514477

[B11] Erttmann S. F. Swacha P. Aung K. M. Brindefalk B. Jiang H. Härtlova A. (2022). The gut microbiota prime systemic antiviral immunity via the cGAS-STING-IFN-I axis. Immunity 55 (5), 847–861. e10. 10.1016/j.immuni.2022.04.006 35545033

[B12] Erturk-Hasdemir D. Oh S. F. Okan N. A. Stefanetti G. Gazzaniga F. S. Seeberger P. H. (2019). Symbionts exploit complex signaling to educate the immune system. Proc. Natl. Acad. Sci. U. S. A. 116 (52), 26157–26166. 10.1073/pnas.1915978116 PMC6936714 31811024

[B13] Furusawa Y. Obata Y. Fukuda S. Endo T. A. Nakato G. Takahashi D. (2013). Commensal microbe-derived butyrate induces the differentiation of colonic regulatory T cells. Nature 504 (7480), 446–450. 10.1038/nature12721 24226770

[B14] Howell K. J. Kraiczy J. Nayak K. M. Gasparetto M. Ross A. Lee C. (2018). DNA methylation and transcription patterns in intestinal epithelial cells from pediatric patients with inflammatory Bowel Diseases differentiate disease subtypes and associate with outcome. Gastroenterology 154 (3), 585–598. 10.1053/j.gastro.2017.10.007 29031501 PMC6381389

[B15] Iwasaki T. Chin W. W. Ko L. (2001). Identification and characterization of RRM-containing coactivator activator (CoAA) as TRBP-interacting protein, and its splice variant as a coactivator modulator (CoAM). J. Biol. Chem. 276 (36), 33375–33383. 10.1074/jbc.M101517200 11443112

[B16] Kang Y. K. Schiff R. Ko L. Wang T. Tsai S. Y. Tsai M. J. (2008). Dual roles for coactivator activator and its counterbalancing isoform coactivator modulator in human kidney cell tumorigenesis. Cancer Res. 68 (19), 7887–7896. 10.1158/0008-5472.CAN-08-1734 18829545 PMC2597518

[B17] Kuhn K. A. Schulz H. M. Regner E. H. Severs E. L. Hendrickson J. D. Mehta G. (2018). Bacteroidales recruit IL-6-producing intraepithelial lymphocytes in the colon to promote barrier integrity. Mucosal Immunol. 11 (2), 357–368. 10.1038/mi.2017.55 28812548 PMC5815964

[B18] Lee J. Y. Tsolis R. M. Bäumler A. J. (2022). The microbiome and gut homeostasis. Science. in press. 10.1126/science.abp9960 35771903

[B19] Li X. Hoeppner L. H. Jensen E. D. Gopalakrishnan R. Westendorf J. J. (2009). Co-activator activator (CoAA) prevents the transcriptional activity of Runt domain transcription factors. J. Cell. Biochem. 108 (2), 378–387. 10.1002/jcb.22263 19585539 PMC3876284

[B20] Maerz J. K. Steimle A. Lange A. Bender A. Fehrenbacher B. Frick J. S. (2018). Outer membrane vesicles blebbing contributes to B. Vulgatus mpk-mediated immune response silencing. Gut Microbes 9 (1), 1–12. 10.1080/19490976.2017.1344810 28686482 PMC5914909

[B21] Martínez-López M. Iborra S. Conde-Garrosa R. Mastrangelo A. Danne C. Mann E. R. (2019). Microbiota sensing by mincle-syk Axis in dendritic cells regulates interleukin-17 and -22 production and promotes intestinal barrier integrity. Immunity 50 (2), 446–461. e9. 10.1016/j.immuni.2018.12.020 30709742 PMC6382412

[B22] Maslowski K. M. Vieira A. T. Ng A. Kranich J. Sierro F. Yu D. (2009). Regulation of inflammatory responses by gut microbiota and chemoattractant receptor GPR43. Nature 461 (7268), 1282–1286. 10.1038/nature08530 19865172 PMC3256734

[B23] Melmed G. Thomas L. S. Lee N. Tesfay S. Y. Lukasek K. Michelsen K. S. (2003). Human intestinal epithelial cells are broadly unresponsive to toll-like receptor 2-dependent bacterial ligands: Implications for host-microbial interactions in the gut. J. Immunol. 170 (3), 1406–1415. 10.4049/jimmunol.170.3.1406 12538701

[B24] Miyauchi E. Shimokawa C. Steimle A. Desai M. S. Ohno H. (2022). The impact of the gut microbiome on extra-intestinal autoimmune diseases. Nat. Rev. Immunol. in press. 10.1038/s41577-022-00727-y 35534624

[B25] Needham B. D. Funabashi M. Adame M. D. Wang Z. Boktor J. C. Haney J. (2022). A gut-derived metabolite alters brain activity and anxiety behaviour in mice. Nature 602 (7898), 647–653. 10.1038/s41586-022-04396-8 35165440 PMC9170029

[B26] Nemet I. Saha P. P. Gupta N. Zhu W. Romano K. A. Skye S. M. (2020). A cardiovascular disease-linked gut microbial metabolite acts via adrenergic receptors. Cell 180 (5), 862–877. e22. 10.1016/j.cell.2020.02.016 32142679 PMC7402401

[B27] Paik D. Yao L. Zhang Y. Bae S. D'Agostino G. D. Zhang M. (2022). Human gut bacteria produce Τ _Η_ 17-modulating bile acid metabolites. Nature 603 (7903), 907–912. 10.1038/s41586-022-04480-z 35296854 PMC9132548

[B28] Park E. M. Chelvanambi M. Bhutiani N. Kroemer G. Zitvogel L. Wargo J. A. (2022). Targeting the gut and tumor microbiota in cancer. Nat. Med. 28 (4), 690–703. 10.1038/s41591-022-01779-2 35440726

[B29] Perez-Riverol Y. Bai J. Bandla C. Hewapathirana S. García-Seisdedos D. Kamatchinathan S. (2022). The PRIDE database resources in 2022: A hub for mass spectrometry-based proteomics evidences. Nucleic Acids Res. 50 (D1), D543–D552. 10.1093/nar/gkab1038 34723319 PMC8728295

[B30] Price A. E. Shamardani K. Lugo K. A. Deguine J. Roberts A. W. Lee B. L. (2018). A map of toll-like receptor expression in the intestinal epithelium reveals distinct spatial, cell type-specific, and temporal patterns. Immunity 49 (3), 560–575. e6. 10.1016/j.immuni.2018.07.016 30170812 PMC6152941

[B31] Rahman M. M. Islam F.-Or-Rashid M. H. Mamun A. A. Rahaman M. S. Islam M. M. (2022). The gut microbiota (microbiome) in cardiovascular disease and its therapeutic regulation. Front. Cell. Infect. Microbiol. 12, 903570. 10.3389/fcimb.2022.903570 35795187 PMC9251340

[B32] Round J. L. Mazmanian S. K. (2010). Inducible Foxp3+ regulatory T-cell development by a commensal bacterium of the intestinal microbiota. Proc. Natl. Acad. Sci. U. S. A. 107 (27), 12204–12209. 10.1073/pnas.0909122107 20566854 PMC2901479

[B33] Sadrekarimi H. Gardanova Z. R. Bakhshesh M. Ebrahimzadeh F. Yaseri A. F. Thangavelu L. (2022). Emerging role of human microbiome in cancer development and response to therapy: Special focus on intestinal microflora. J. Transl. Med. 20 (1), 301. 10.1186/s12967-022-03492-7 35794566 PMC9258144

[B34] Schluter J. Peled J. U. Taylor B. P. Markey K. A. Smith M. Taur Y. (2020). The gut microbiota is associated with immune cell dynamics in humans. Nature 588 (7837), 303–307. 10.1038/s41586-020-2971-8 33239790 PMC7725892

[B35] Sender R. Fuchs S. Milo R. (2016). Are we really vastly outnumbered? Revisiting the ratio of bacterial to host cells in humans. Cell 164 (3), 337–340. 10.1016/j.cell.2016.01.013 26824647

[B36] Shiratsuchi G. Takaoka K. Ashikawa T. Hamada H. Kitagawa K. (2015). RBM14 prevents assembly of centriolar protein complexes and maintains mitotic spindle integrity. EMBO J. 34 (1), 97–114. 10.15252/embj.201488979 25385835 PMC4291483

[B37] Sobhani I. Bergsten E. Couffin S. Amiot A. Nebbad B. Barau C. (2019). Colorectal cancer-associated microbiota contributes to oncogenic epigenetic signatures. Proc. Natl. Acad. Sci. U. S. A. 116 (48), 24285–24295. 10.1073/pnas.1912129116 31712445 PMC6883805

[B38] Stefan K. L. Kim M. V. Iwasaki A. Kasper D. L. (2020). Commensal microbiota modulation of natural resistance to virus infection. Cell 183 (5), 1312–1324. e10. 10.1016/j.cell.2020.10.047 33212011 PMC7799371

[B39] Sugi Y. Takahashi K. Kurihara K. Nakata H. Narabayashi Y. Hamamoto M. (2016). Post-transcriptional regulation of toll-interacting protein in the intestinal epithelium. PLoS One 11 (10), e0164858. 10.1371/journal.pone.0164858 27741296 PMC5065231

[B40] Sujino T. London M. Hoytema van Konijnenburg D. P. Rendon T. Buch T. Silva H. M. (2016). Tissue adaptation of regulatory and intraepithelial CD4⁺ T cells controls gut inflammation. Science 352 (6293), 1581–1586. 10.1126/science.aaf3892 27256884 PMC4968079

[B41] Sultan S. El-Mowafy M. Elgaml A. Ahmed T. A. E. Hassan H. Mottawea W. (2021). Metabolic influences of gut microbiota dysbiosis on inflammatory bowel disease. Front. Physiol. 12, 715506. 10.3389/fphys.2021.715506 34646151 PMC8502967

[B42] Takahashi K. Sugi Y. Hosono A. Kaminogawa S. (2009). Epigenetic regulation of TLR4 gene expression in intestinal epithelial cells for the maintenance of intestinal homeostasis. J. Immunol. 183 (10), 6522–6529. 10.4049/jimmunol.0901271 19846881

[B43] Takahashi K. Sugi Y. Nakano K. Kobayakawa T. Nakanishi Y. Tsuda M. (2020). Regulation of gene expression through gut microbiota-dependent DNA methylation in colonic epithelial cells. Immunohorizons 4 (4), 178–190. 10.4049/immunohorizons.1900086 32295802

[B44] Takahashi K. Sugi Y. Nakano K. Tsuda M. Kurihara K. Hosono A. (2011). Epigenetic control of the host gene by commensal bacteria in large intestinal epithelial cells. J. Biol. Chem. 286 (41), 35755–35762. 10.1074/jbc.M111.271007 21862578 PMC3195625

[B45] Tan A. H. Lim S. Y. Lang A. E. (2022). The microbiome-gut-brain Axis in Parkinson disease – from basic research to the clinic. Nat. Rev. Neurol. 18, 476–495. in press. 10.1038/s41582-022-00681-2 35750883

[B46] Verreman K. Baert J. L. Verger A. Drobecq H. Ferreira E. de Launoit Y. (2011). The coactivator activator CoAA regulates PEA3 group member transcriptional activity. Biochem. J. 439, 469–477. 10.1042/BJ20110728 21736557

[B47] Wei G. Z. Martin K. A. Xing P. Y. Agrawal R. Whiley L. Wood T. K. (2021). Tryptophan-metabolizing gut microbes regulate adult neurogenesis via the aryl hydrocarbon receptor. Proc. Natl. Acad. Sci. U. S. A. 118 (27), e2021091118. 10.1073/pnas.2021091118 34210797 PMC8271728

[B48] Yuan M. Eberhart C. G. Kai M. (2014). RNA binding protein RBM14 promotes radio-resistance in glioblastoma by regulating DNA repair and cell differentiation. Oncotarget 5 (9), 2820–2826. 10.18632/oncotarget.1924 24811242 PMC4058047

